# Transcriptome Analysis Explored the Differential Genes’ Expression During the Development of the *Stropharia rugosoannulata* Fruiting Body

**DOI:** 10.3389/fgene.2022.924050

**Published:** 2022-06-29

**Authors:** Cui Wang, Xunjie Zhang, Zhiheng Zeng, Feifei Song, Zhen Lin, Liangjun Chen, Zhixin Cai

**Affiliations:** ^1^ Department of Health and Food, Fujian Vocational College of Bioengineering, Fuzhou, China; ^2^ Institute of Edible Mushroom, Fujian Academy of Agricultural Sciences, Fuzhou, China

**Keywords:** *Stropharia rugosoannulata*, fruiting body, growth and development, alternative splicing events, DEGs

## Abstract

*Stropharia rugosoannulata* (*S*. *rugosoannulata*) is a fungus with great edible and nutritional values; however, the development mechanism of its fruiting body has not been studied. Thus, this study aimed to analyze the differentially expressed genes (DEGs) in four stages; primordia stage (Sra1), young mushroom stage (Sra2), picking stage (Sra3), and opening umbrella stage (Sra4). Therefore, total RNA was extracted for further RNA-sequencing analysis. In three pairwise comparison groups (PCGs), Sra1 vs. Sra2, Sra2 vs. Sra3, and Sra3 vs. Sra4, a total of 3,112 DEGs were identified among the three PCGs. A GO analysis of the DEGs showed that there were 21 terms significantly enriched in Sra1 vs. Sra2 PCG. There was no significantly enriched GO term in the other two PCGs. Furthermore, KEGG pathway analysis showed that these DEGs were mainly enriched in glucose and amino acid metabolisms. Moreover we found that intron retention (IR) and the alternative 3′ splice site (A3SS) accounted for more than 80%. The development of the *S*. *rugosoannulata* fruiting body mainly involved glucose and amino acid metabolisms. IR and A3SS were the two main types of ASE, which played an important role in the development and maturation of the *S*. *rugosoannulata* fruiting body.

## Introduction

*Stropharia rugosoannulata* (*S*. *rugosoannulata*, also known as wine cap stropharia in English), is an edible fungus of Stropharia in *Strophariaceae* with high medicinal and nutritional values (Liu et al., 2020). As an environment-friendly fungus, *S*. *rugosoannulata* is mainly cultivated with straw and does not consume forest resources. Therefore, it is recommended by the Food and Agriculture Organization as a good food source for developing countries (Li et al., 2022). *S*. *rugosoannulata* is consumed in large quantities and cultivated in many countries due to its superior taste and pharmacological activity (Yang et al., 2022). Other studies have shown that *S*. *rugosoannulata* could degrade environmental pollutants (bisphenol A, 2,4,6-trinitrotoluene, dibenzo-p-dioxins, and dibenzofurans) and plays an important role in soil remediation (Weiss et al., 2004; Kabiersch et al., 2011; Anasonye et al., 2014). At present, only a few studies have focused on the cultivation techniques and chemical constituents of *S*. *rugosoannulata* (Gong et al., 2018; Liu et al., 2019), and the regulation mechanism of *S*. *rugosoannulata* growth and development remains almost unknown.

Fruiting body, as the main edible part of edible fungi, is also an important structure for the reproductive growth of the fungi, and its development and maturation are regulated by a variety of development-related genes and signal pathways (Teichert et al., 2020). For example, in the development stage of the *Cordyceps militaris* fruiting body, the transcription level of the CmVVD gene increased significantly, and the deletion of CmVVD led to the obstacle of fruiting body development (Zhang et al., 2020). During the development of the fruiting body of *Flammulina velutipes*, dikaryotic Fvclp1 gene knockdown resulted in almost no development of the fruiting body, while the fruiting body with Fvclp1 overexpression developed faster and had longer stipes (Lyu et al., 2021). Therefore, it is of great significance to explore gene expression patterns and excavate potential functional genes to clarify the molecular mechanism of *S*. *rugosoannulata* fruiting body development. Recently, the molecular mechanism of the fruiting body formation and development in edible fungi has made great progress, due to the innovation of transcriptome sequencing technology. For example, Wang et al. explored the molecular mechanism of the growth of the *Lentinula edodes* fruiting body from the early bud stage to the intermediate developing stage to the fully developed stage by high-throughput Illumina RNA sequencing. The results showed that the differentially expressed genes (DEGs) were mainly involved in purine and fatty acid metabolism during the growth and development of the fruiting bodies (Wang et al., 2018). Wan et al. used whole-genome and transcriptome sequencing to reveal the mechanism of lignocellulose degradation and fruiting body development of *Phlebopus portentosus*. The results show that 126 genes were highly related to fruiting body development, and these genes were mainly enriched in DNA replication, proteasome, and MAPK signaling pathways (Wan et al., 2021). Zhang et al. analyzed the transcriptome of *Hypsizygus marmoreus* at four different developmental stages and found that nitrogen starvation was one of the most important factors to promote fruiting body maturation, and nitrogen metabolism and the mTOR signaling pathway were related to this process (Zhang et al., 2015).

Given the importance of the *S*. *rugosoannulata* fruiting body development mechanism in functional gene screening, genetic breeding, and cultivation, this study aimed to explore the DEGs in *S. Rugosoannulata* fruiting body at different development stages by using RNA-sequencing technology, and analyze the underlying mechanism, which provided a reference for the development of molecular markers, functional gene cloning, and genetic engineering improvement.

## Materials and Methods

### Collection of *S*. *rugosoannulata* Fruiting Body Samples at Four Developmental Stages

In this study, the mushroom production experiment of *S*. *rugosoannulata* was carried out in the annual cultivation room of Edible Fungi Research Institute of Fujian Academy of Agricultural Sciences. The environmental conditions for mushroom production were set as follows: temperature, 16–18°C; relative humidity, 90–95%; and illumination, 100–200 lux (illumination for 2 h per day). *S*. *rugosoannulata* also belongs to *Basidiomycetes*. The fruiting body development of *Basidiomycetes* was generally divided into the primordia stage (Sra1), young mushroom stage (Sra2), picking stage (Sra3), and opening umbrella stage (Sra3). The development differentiation of these four stages was obvious and easy to distinguish. Therefore, the fresh *S*. *rugosoannulata* fruiting body tissues from the four developmental stages were collected, and the same part of the fungus cap was collected from each tissue, which was frozen using liquid nitrogen and then stored in a −80°C refrigerator, and three samples were collected at each stage as replicates.

### Total RNA Extraction and Quality Detection

Total RNA of *S*. *rugosoannulata* fruiting body samples was extracted using TRIzol®Reagent (Invitrogen) according to the manufacturer’s instructions. The purity and concentration of total RNA were examined by NanoDrop 2000, and the RNA integrity number value of total RNA was detected by Agilent2100. The qualified total RNA was sequenced through the Illumina next-generation high-throughput sequencing platform.

### cDNA Library Construction and RNA Sequencing

For qualified RNA samples, mRNA was enriched with magnetic beads containing Oligo (dT) and then synthesized cDNA by reverse transcription. Under the action of reverse transcriptase, the first cDNA strand was synthesized with random six base primers. The second cDNA strand was synthesized by adding a buffer, DNA polymerase I, and dNTPs. Then, double-stranded cDNA was purified using AMPure XP Beads. The purified double-stranded cDNA was treated with end repaired mix; A-bases and splice were also added. The AMPure XP Beads were used to select the fragment size of the double-strand cDNA, and finally PCR amplification was performed to construct cDNA libraries. The Illumina sequencing platform was used for library sequencing. The library preparations were sequenced on an Illumina Novaseq 6000platform by the Beijing Allwegene Technology Company Limited (Beijing, China) and paired-end 150bp reads were generated.

### Quality Control of Raw Data and Reference Mapping

Trimmomatic software (version 0.33) was used to conduct quality control on the raw data to ensure the accuracy of the analysis. Reads containing adapter, N ratio greater than 10% (N indicated that base could not be determined), and having low quality were removed to obtain high-quality sequences for the subsequent analysis. Sequence reads were mapped to the reference genome GCA_003314255.1 using Spliced Transcripts Alignments to a Reference (STAR, version 2.5.2b) software.

### DEGs Screening

HTSeq software (version 0.6.1p1) was used to analyze the gene expression levels, using the union mode. The expression levels of individual genes and the number of genes at different expression levels were presented in the results. Then we used the DESeq2 package in R software (version 3.5.2) to analyze the DEGs between different groups. The threshold of DEG screening was |log_2_ (foldchange)| > 1 and *p* < 0.05.

### Functional Enrichment Analysis

For the DEGs, we used “clusterProfiler” function package in R software (version 3.5.2) to conduct the enrichment analysis on GO terms and KEGG pathways. The significantly enriched GO term and KEGG pathway were screened with *p* value < 0.05 after correction by the Benjamini and Hochberg (BH) method.

### Differential Alternative Splicing Analysis

Differential alternative splicing events from RNA-sequencing data were assessed using rMATS (version 4.0.2). And then, *p* value was calculated using the likelihood-ratio test to represent the difference of the inclusion level between the two groups of samples. There were five types of differential alternative splicing events detected by rMATS, including skipped exon (SE), alternative 5′ splice site (A5SS), alternative 3′ splice site (A3SS), mutually exclusive exons (MXE), and intron retention (IR) (Hooper et al., 2020).

### Statistical Analysis

All statistical analysis was completed using R software (version 3.5.2). *p* < 0.05 was regarded as statistically.

## Results

### Statistics of Raw Data

After removing the low-quality sequences with Trimmomatic software, the sequencing amount of each sample was counted, and the results are shown in Supplementary Table S1. As listed in Supplementary Table S1, the sequencing data Q20 (base correct recognition rate > 99%) were all more than 97%, Q30 (base correct recognition rate > 99.9%) were all more than 93%, and the GC content were all less than 54%, indicating that the sequencing quality met the subsequent analysis standards (Supplementary Figures S1A–L showed the distribution of the base error rate, and Supplementary Figures S2A–L showed the distribution of A/T/C/G base content).

### The Results of Reference Mapping

STAR software was used to map the processed data with the reference genome GCA_003314255.1, and a statistical analysis was performed on the exon, intron, and intergenic regions of the reference sequence according to reads (Supplementary Table S2). As shown in Supplementary Figures S3A–L, most reads were mapped to exon, but rarely to the intron region. In the species with relatively complete genome annotation, the reads mapped to exon had the highest content, and the reads mapped to the intron region were mainly caused by the residue of pre-mRNA and intron retention events during the variable shearing process. The presence of reads to intergenic was generally due to incomplete genomic annotations.

### DEGs Screening at Four Different Development Stages of Fruiting Body

**FIGURE 1:**
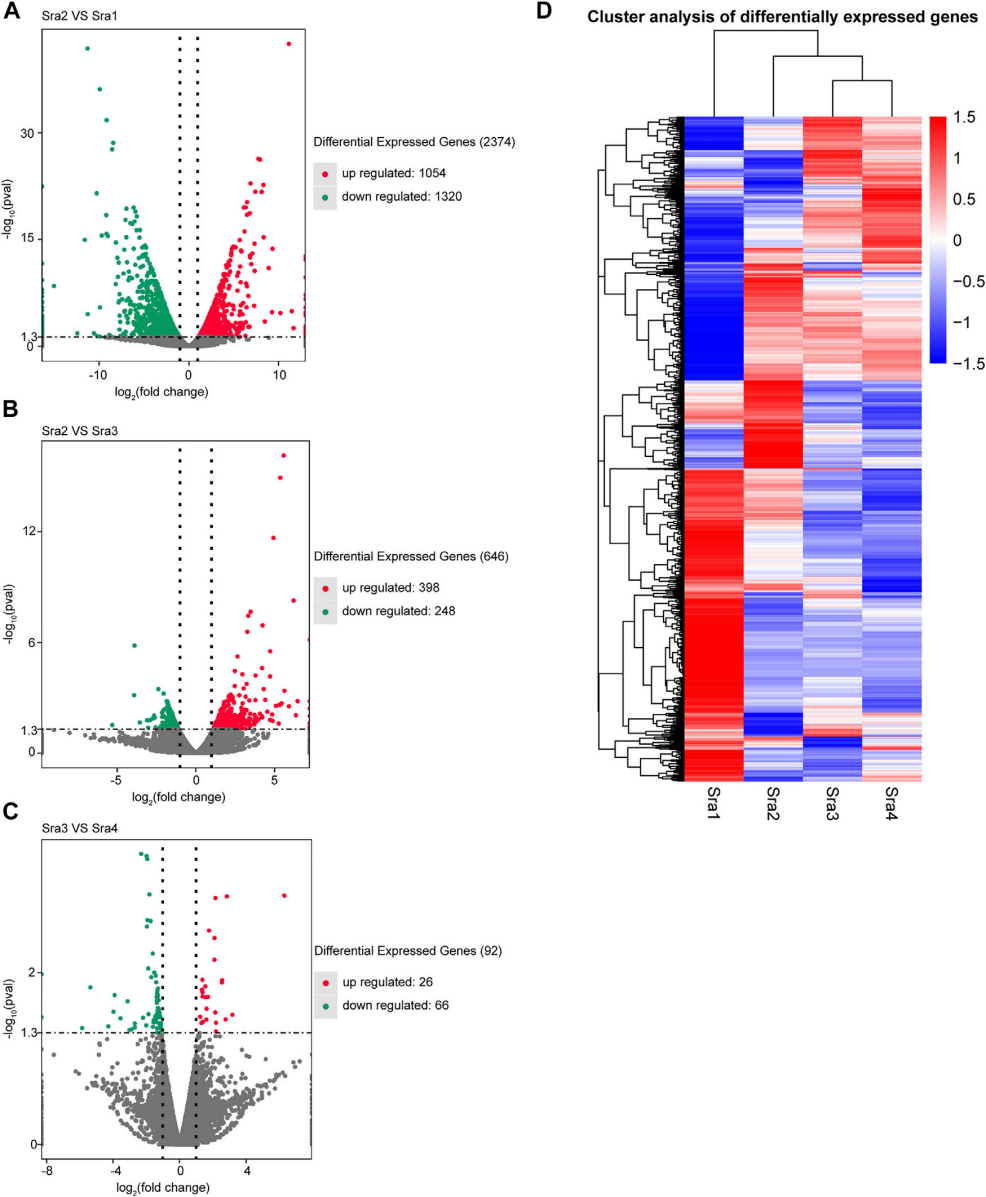
Screening of DEGs. **(A–C)** Volcano map of DEGs in each PCG, **(A)** Sra1 vs. Sra2 PCG; **(B)** Sra2 vs. Sra3 PCG; and **(C)** Sra3 vs. Sra4 PCG. The horizontal axis represents the variation of gene expression multiple in different samples, and the vertical axis represents the statistical significance of the variation of the gene expression level. Genes with a significant differential expression were represented by red dots (up-regulated) and green dots (down-regulated). Genes with insignificant differential expressions were represented by gray dots. **(D)** DEG clustering diagram of the 4 groups of samples.

In this study, a transcriptome analysis was performed on samples from four developmental stages of *S*. *rugosoannulata* fruiting bodies. We divided the four stages of Sra1–4 into three pairwise comparison groups (PCGs) according to the two successive development periods as follows: Sra1 vs. Sra2 group, Sra2 vs. Sra3 group, and Sra3 vs. Sra4 group. A total of 3,112 DEGs were identified among the three PCGs (as listed in Supplementary Table S3), compared with Sra1, there were 2,374 DEGs in Sra2, including 1,054 up-regulated genes and 1,320 down-regulated genes (Figure 1A). Compared with Sra3, there were 646 DEGs in Sra2, including 398 up-regulated genes and 248 down-regulated genes (Figure 1B). Compared with Sra4, there were 92 DEGs in Sra3, including 26 up-regulated genes and 66 down-regulated genes (Figure 1C). A hierarchical cluster analysis was carried out on the expression levels of differential genes, and the differential genes were divided into several clusters. As shown in Figure 1D, genes in the same cluster had similar change trends of expression levels under different treatment conditions.

### GO and KEGG Classification

**FIGURE 2:**
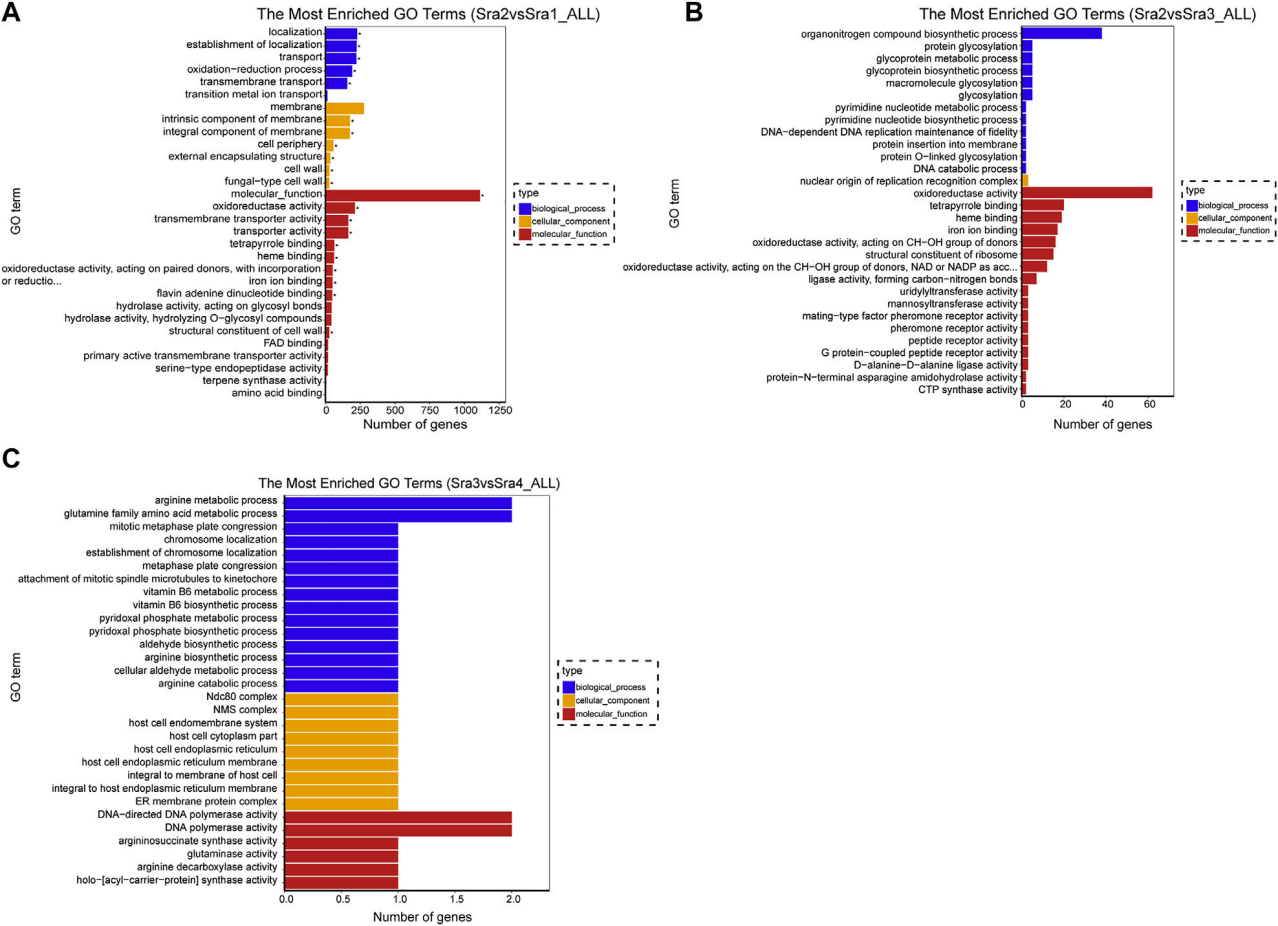
GO classification of DEGs. **(A–C)** GO enrichment histogram of three PCGs. **(A)** Sra1 vs. Sra2 PCG; **(B)** Sra2 vs. Sra3 PCG; and **(C)** Sra3 vs. Sra4 PCG. The vertical axis was the name of the enriched GO term, and the horizontal axis was the number of DEGs in the corresponding term. Different colors were used to distinguish biological processes, cell components, and molecular functions, and “*” represented a significantly enriched GO term.

**FIGURE 3:**
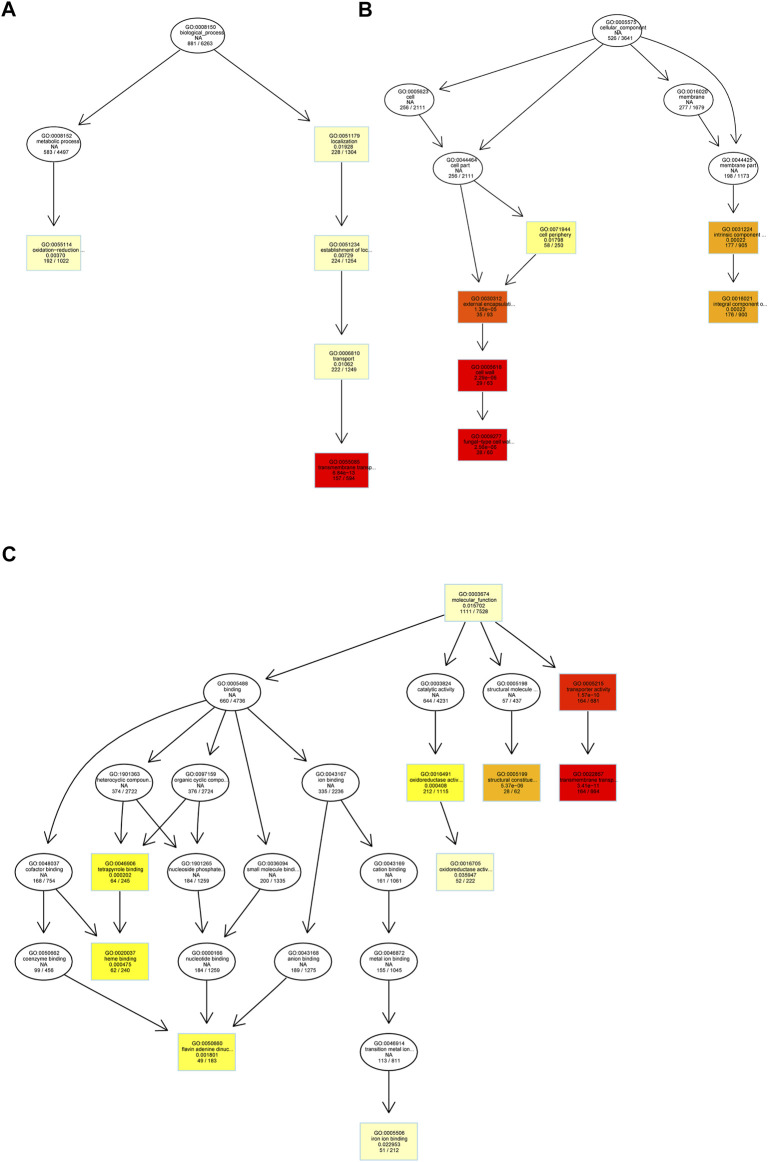
Directed acyclic graph of the top GO terms in Sra1 vs. Sra2 PCG. **(A)** Biological process; **(B)** cell component; and **(C)** molecular function. Each node represents a GO term, and the contents displayed on each node from top to bottom are as follows: GO term ID, description, *p* value after correction, and the number of DEGs under the GO term. The depth of color represents the degree of enrichment, and the darker the color, the more significant the degree of enrichment.

To better understand the biological processes occurring in *S*. *rugosoannulata* fruiting body growth, we used the “clusterProfiler” function package in R software to conduct the enrichment analysis of the DEGs in GO terms and KEGG pathways. A GO enrichment analysis of DEGs in three PCGs showed that there were 21 terms significantly enriched in Sra1 vs. Sra2 PCG (Figure 2A), including 5 biological process terms (transmembrane transport, oxidation-reduction process, establishment of localization, transport, and localization), 6 cellular component terms (intrinsic component of membrane, integral component of membrane, cell periphery, etc.), and 10 molecular function terms (molecular function, oxidoreductase activity, transmembrane transporter activity, etc.). There was no significantly enriched GO term in the other two PCGs (Figures 2B,C). We also established a directed acyclic graph for top GO terms and found that the top GO terms were mainly involved in transmembrane transport (GO: 0055085) (Figure 3A), fungal type cell wall (GO: 0009277) (Figure 3B), and transmembrane transporter activity (GO: 0022857) (Figure 3C).

**FIGURE 4:**
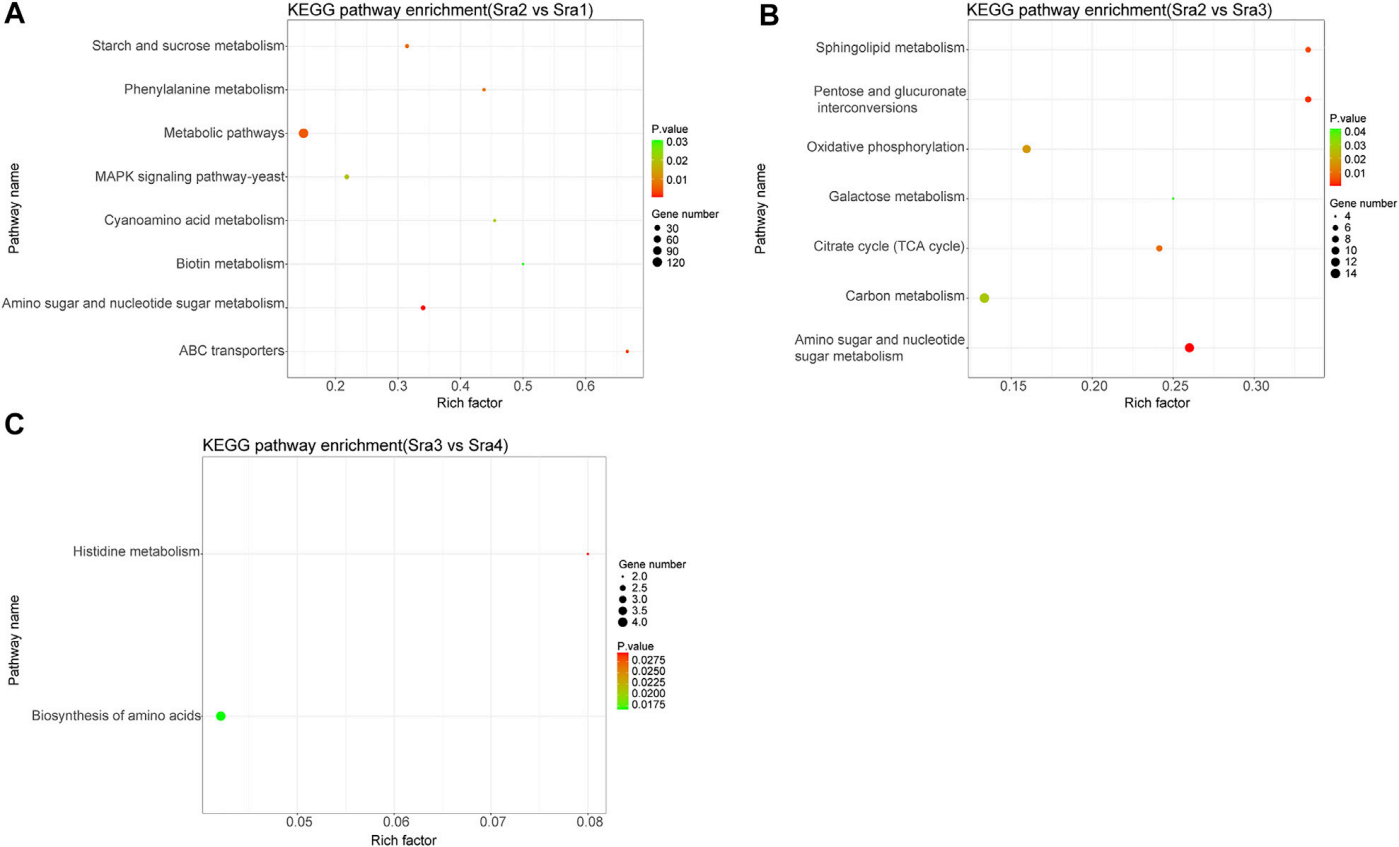
KEGG pathway enrichment analysis of three PCGs. **(A)** Sra1 vs. Sra2 PCG; **(B)** Sra2 vs. Sra3 PCG; and **(C)** Sra3 vs. Sra4 PCG. The vertical axis represents the pathway, the horizontal axis represents rich factor, the size of dots indicates the number of differentially expressed genes in the pathway, and the color of dots corresponds to different *p* value ranges.

Further KEGG pathway enrichment analysis showed that DEGs in the Sra2 vs. Sra1 group were significantly enriched in amino sugar and nucleotide sugar metabolisms, ATP-binding cassette (ABC) transporters, and metabolic pathways (Figure 4A). DEGs in Sra2 vs. Sra3 group were significantly enriched in amino sugar and nucleotide sugar metabolisms, pentose and glucuronate interconversions, and sphingolipid metabolisms (Figure 4B). DEGs in Sra3 vs. Sra4 groups were significantly enriched in the biosynthesis of amino acids and histidine metabolism (Figure 4C). It has been reported that *S*. *rugosoannulata* was rich in amino acids and polysaccharides (Liu et al., 2019; Liu et al., 2020), and active amino acids and glucose metabolic pathways might contribute to the accumulation of these substances. All enriched GO terms and KEGG pathways are shown in Supplementary Tables S4, S5.

### Characteristics of Differential Alternative Splicing Events in Different Developmental Stages

**FIGURE 5:**
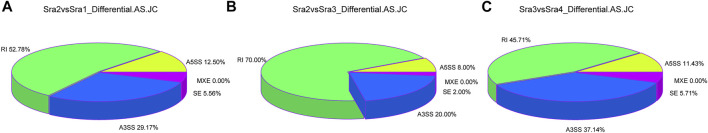
Alternative splicing events at the different stages of *S*. *rugosoannulata* fruiting body. **(A–C)** Differential alternative splicing events in Sra1 vs. Sra2, Sra2 vs. Sra3, and Sra3 vs. Sra4, respectively. IR: intron retention, A5SS: alternative 5′ splice site, A3SS: alternative 3′ splice site, MXE: mutually exclusive exons, and SE: skipping exon.

Alternative splicing was one of the main mechanisms for protein diversity (Yuan et al., 2021). We used rMATS to compare the difference in the inclusion level between the two groups of *S*. *rugosoannulata* samples, evaluate the change of the corresponding inclusion level in the two samples, and obtain the distribution map of the differential alternative splicing events in each comparison group (Figures 5A–C). In the Sra1 vs. Sra2 PCG, the alternative splicing events of IR, A5SS, A3SS, and SE accounted for 52.78, 12.50, 29.17, and 5.56%, respectively (Figure 1A). In the Sra2 vs. Sra3 PCG, the alternative splicing events of IR, A5SS, A3SS, and SE accounted for 70.00, 8.00, 20.00, and 2.00, respectively (Figure 5B). In the Sra3 vs. Sra4 PCG, the alternative splicing events of IRI, A5SS, A3SS, and SE accounted for 45.71, 11.43, 37.14, and 5.71%, respectively (Figure 5C).

We found that IR and A3SS accounted for more than 80% from the primordia stage to the picking stage of *S*. *rugosoannulata* fruiting body, and the proportion of IR from the young mushroom stage to the picking stage was the highest and then showed a downward trend, while A3SS increased in the later stage of fruiting body maturation, indicating that these two alternative splicing events were likely to be mainly involved in the regulation of *S*. *rugosoannulata* fruiting body development and maturation.

## Discussion

In this study, we analyzed DEGs and differential alternative splicing events related to the growth and development of *S*. *rugosoannulata* fruiting bodies by comparing the transcriptome data of *S*. *rugosoannulata* fruiting bodies at the primordia stage, young mushroom stage, picking stage, and opening umbrella stage, providing clues for further understanding the regulation mechanism of *S*. *rugosoannulata* development.

*S*. *rugosoannulata* is a popular edible fungus with beneficial healthcare value, so it is of great significance to analyze the functional genes in its growth and development process for quality improvement. To our knowledge, this study was the first systematic transcriptome study of *S*. *rugosoannulata* fruiting bodies at different developmental stages. At different stages of fruiting body development, there were significant differences in gene transcription levels. We noticed that the number of DEGs was the largest from the primordia stage to the young mushroom stage, while it was significantly reduced from the picking stage to the opening umbrella stage, which was consistent with the study of Tong et al. (2020). In the four stages of the fruiting body development, DEGs were involved in the biological processes of transmembrane transport, oxidation–reduction process, fungal cell wall, etc. In the early stages of growth, genes were enriched in the basic substance synthesis pathway and transmembrane transport, indicating that the early *S*. *rugosoannulata* fruiting body was vigorous in growth and development with an active metabolism and increased demand for energy and protein (Tong et al., 2020). The active transmembrane transport process facilitated the exchange of materials between cells (Tomkins et al., 2021), thereby promoting the development of fruiting bodies. While in the later stage of growth, DEGs significantly decreased to only 92, which might be attributed to the slow growth after fruiting body maturation (Wang et al., 2020), and DEGs were enriched in synthesis pathways of secondary metabolites. Activated pathways of sugar and amino acid syntheses and metabolisms made *S*. *rugosoannulata* fruiting bodies rich in polysaccharides and amino acids (Liu et al., 2019; Liu et al., 2020). The DEGs were enriched in the oxidation–reduction process during early fruiting body development, which was also of interest to us. Previous studies have shown that reactive oxygen species (ROS) played an important role in the development of fungi. High concentrations of ROS were considered toxic, while maintaining an appropriate level of ROS had a positive role in regulating fungal development, such as promoting mycelial branching and primordium formation of *Ganoderma lucidum* (Li et al., 2015). This evidence suggested that ROS homeostasis might affect the development of *S*. *rugosoannulata* fruiting bodies by regulating the antioxidant enzyme activity and ROS level.

Alternative splicing referred to the transcription process in which two or more mature mRNAs were generated from the same pre-mRNA through different splicing methods, which play an important role in regulating the growth and development of plants as well as responses to environmental stress (Sun and Xiao, 2015). For example, the genome-wide analysis of *Physcomitrella patens* by Chang et al. showed that high temperature caused nearly 50% of the expressed genes to develop alternative splicing, in which the proportion of IR decreased and SE dominated, indicating that alternative splicing had differential regulation in response to heat shock (Chang et al., 2014). Tong et al. demonstrated that IR was the main event in the whole growth and development of *Ophiocordyceps sinensis* (Tong et al., 2020). Yan et al. found that Vv-sod genes possessed two alternative splicing events of IR and A3SS in the development process of *Volvariella volvacea*, and consequently several potential Cu-Znsod1 and Mnsod1/2 amino acid sequences with different lengths and sequence motifs could be obtained by random combinations of alternative splicing events (Yan et al., 2016). These studies revealed the important role of alternative splicing events in gene expression and protein diversity. In our present study, we found that the proportion of IR first increased and then decreased, while the proportion of A3SS increased gradually, and the total proportion of IR and A3SS exceeded 80% in each stage. These results indicate that these two kinds of alternative splicing events were mainly involved in regulating pre-mRNA to produce different mature spliceosomes, regulating the expression of genes in cells, and then affecting the function of the encoded proteins (Laloum et al., 2018). However, which genes undergo alternative splicing and their influence on *S*. *rugosoannulata* fruiting body development were not revealed in this study, which will be focused on in future studies.

## Conclusion

In this study, based on the RNA-sequencing data of four growth and development stages of the *S*. *rugosoannulata* fruiting body, we systematically identified the DEGs at each stage and analyzed their biological functions. The pre-mRNA alternative splicing atlas of the four stages was further constructed, and the characteristics of alternative splicing events among different stages were also identified. We found that the development of the *S*. *rugosoannulata* fruiting body was mainly involved in biosynthesis, glucose metabolism, and amino acid metabolism. IR and A3SS were the two most important types of alternative splicing, which played an important role in the development and maturation of *S*. *rugosoannulata* fruiting bodies. This study was the first to describe the transcriptome dynamics during the development of *S*. *rugosoannulata* fruiting bodies from a gene and pre-mRNA splicing aspect. Our work provided clues for further exploring the molecular mechanism of *S*. *rugosoannulata* fruiting body development.

## Data Availability

The raw data supporting the conclusion of this article will be made available by the authors, without undue reservation.
