# Supplementary figures and images for: Transcriptome Analysis Explored the Differential Genes’ Expression During the Development of the Stropharia rugosoannulata Fruiting Body
Source: Front Genet. 2022 Jun 29;13:924050. doi: 10.3389/fgene.2022.924050 (PMC9318406; doi:10.3389/fgene.2022.924050)

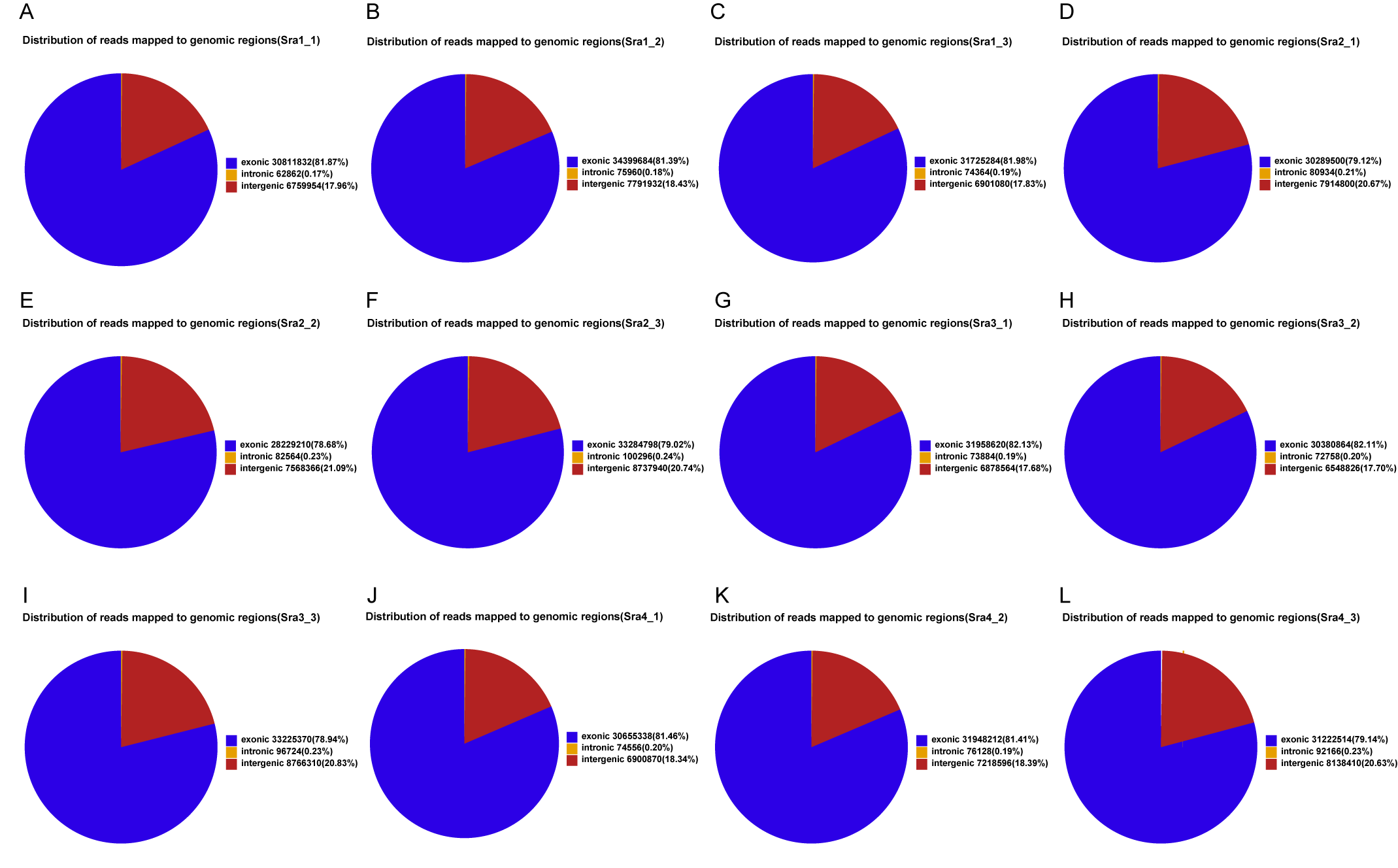

Supplement: Supplementary file 3 [file Image3.TIF]

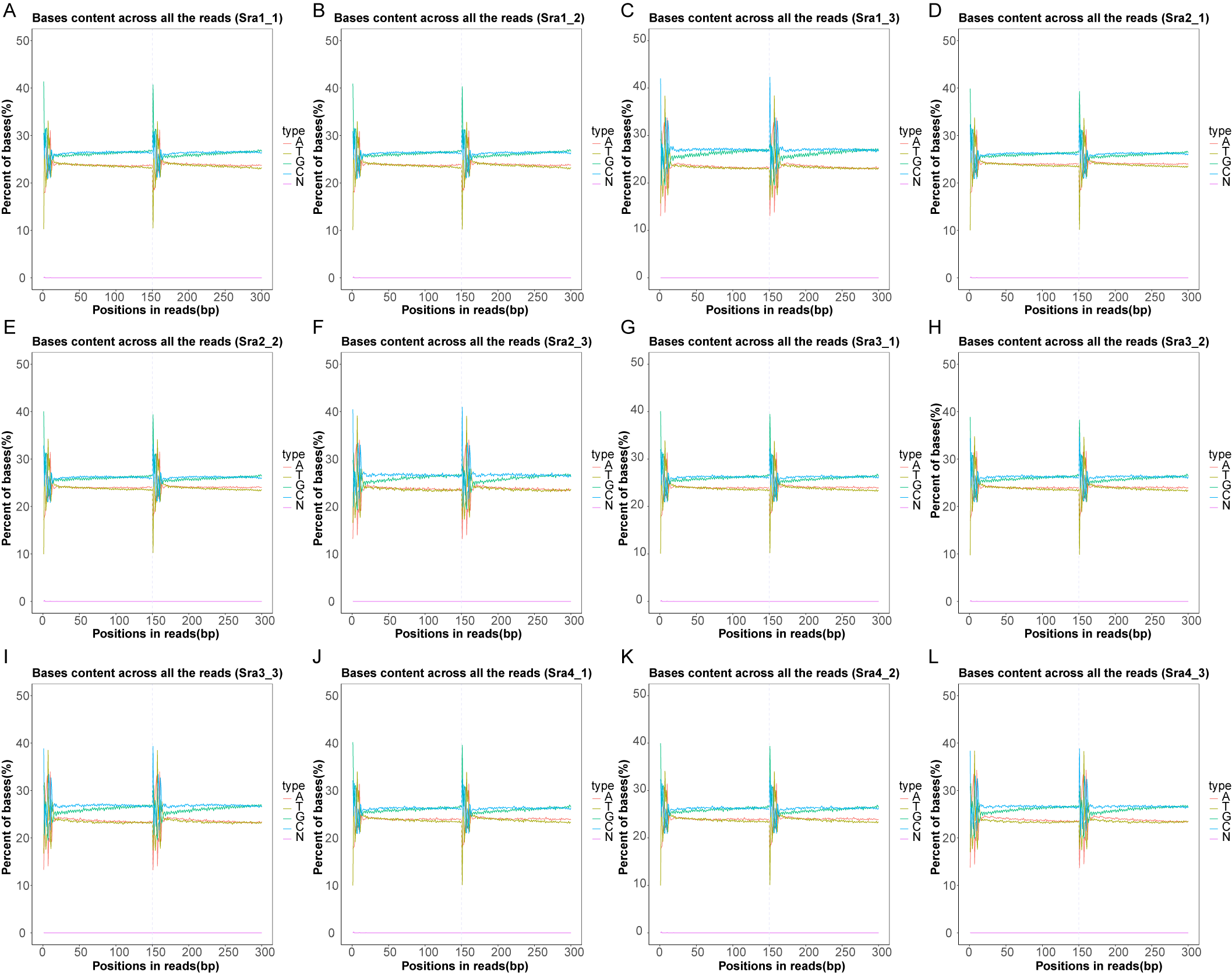

Supplement: Supplementary file 4 [file Image2.TIF]

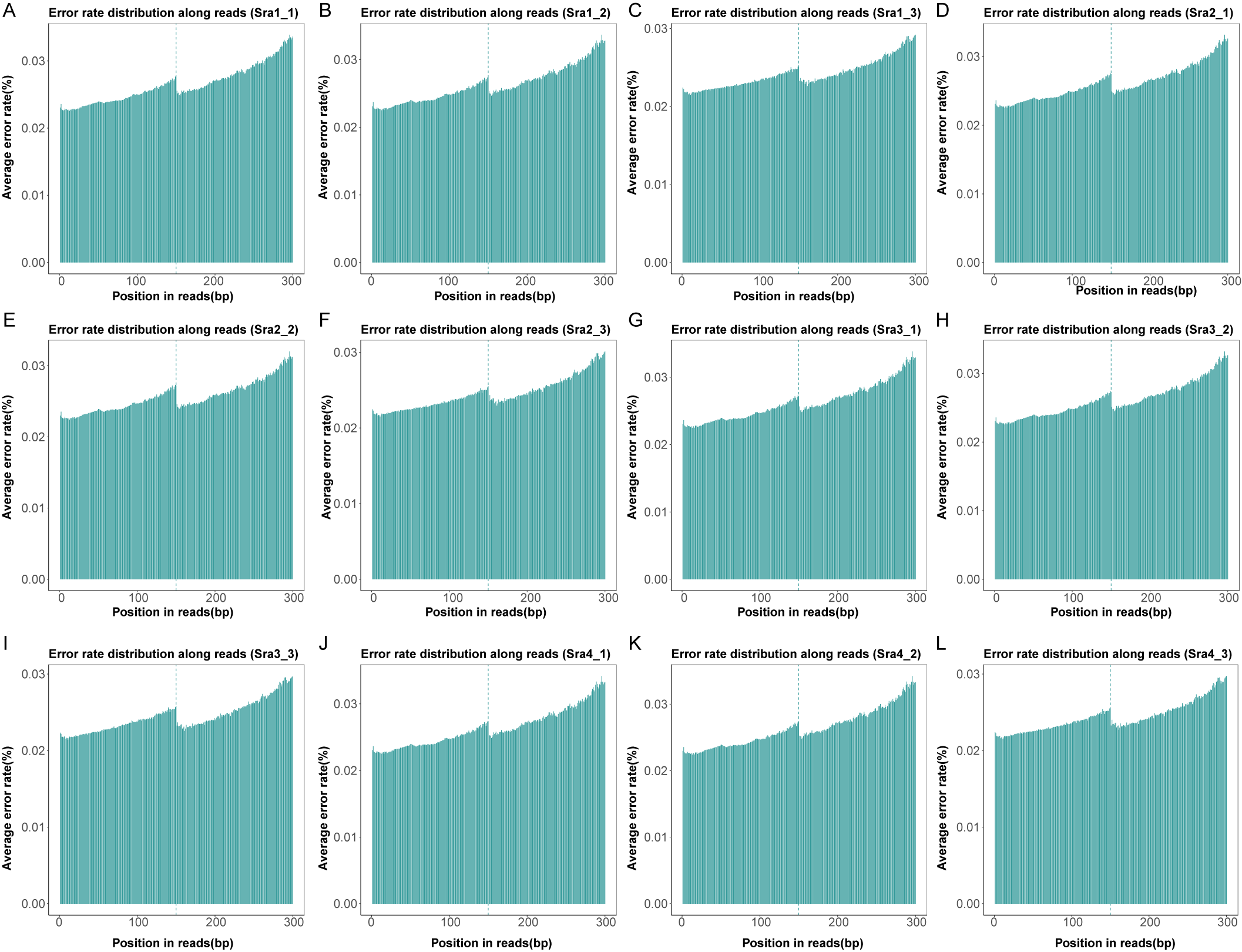

Supplement: Supplementary file 5 [file Image1.TIF]
